# Endoscopic Intermuscular Dissection – Short-term Outcomes

**DOI:** 10.1055/a-2882-1469

**Published:** 2026-06-23

**Authors:** Lisa Birzle, Alanna Ebigbo, Sandra Nagl, Andreas Probst, Mousa Ayoub, Irene Kleinlein, Helmut Messmann

**Affiliations:** 1 Department of Gastroenterology 39694 University Hospital AugsburgAugsburgBavariaGermany; 2 Department of Gastroenterology 91789 Katholisches Klinikum Bochum Sankt Josef-HospitalBochumNordrhein-WestfalenGermany; 3 Institute for Pathology and Molecular Diagnostics 39694 University Hospital AugsburgAugsburgBYGermany

**Keywords:** endoscopy lower GI tract, colorectal cancer, diagnosis and imaging (inc chromoendoscopy, NBI, iSCAN, FICE, CLE, ...), endoscopic resection (polypectomy, ESD, EMRc, ...)

## Abstract

**Introduction** To evaluate the feasibility and outcomes of endoscopic intermuscular dissection (EID), we present a case series of patients with suspected deep submucosal invasive T1 rectal cancers based on pre-interventional assessment. The aim is to describe the potential role of EID within risk-adapted treatment strategies for early rectal cancer.

**Methods** EID was performed in 16 patients at the University Hospitals Augsburg and Bochum. En bloc resection was achieved in all cases. In six patients, the resection site was closed using the Olympus SutuArt hand-suturing device (Olympus, Tokyo, Japan)

**Results** All lesions were resected en bloc without major complications. Histopathology confirmed low-grade adenocarcinomas with deep submucosal invasion, low tumor budding, and no lymphovascular invasion in six cases, and superficial submucosal invasion without high-risk histologic features in one case. One pretreated adenocarcinoma showed low-grade T1 morphology with intermediate tumor budding and perineural invasion. One case was a poorly differentiated (G3) T2 carcinoma. Additional findings included tubular adenomas with high-grade dysplasia ( *n* = 3), low-grade dysplasia ( *n* = 2), one neuroendocrine tumor, and one vascular malformation. Fourteen resections had clear margins. In one deep submucosal invasive cancer (D-SMIC) case, an R1 deep margin was presumed due to specimen disruption. Subsequent transanal full-thickness resection showed no residual tumor. In one pretreated case, involvement of the deep resection margin resulted in an R1 classification.

**Conclusion** This case series demonstrates that EID is a feasible and promising approach for managing rectal D-SMIC. Further data are needed to refine patient selection and strengthen its role as an organ-preserving alternative to radical surgery.

## Introduction

Current guidelines for T1 rectal cancer management consider several pathologic features as critical risk factors for lymph node metastases (LNM), including lymphovascular invasion (LVI), high-grade tumor budding (TB), poor differentiation (PD), and deep submucosal invasion (DSI). Until now, DSI has been defined as exceeding an invasion depth of more than 1000 μm; however, recent studies suggest that its predictive value has likely been overestimated. 1

Current data and metanalyses raise the question of whether patients with suspected DSI might benefit from a less invasive alternative to surgery, an approach like endoscopic intermuscular dissection (EID), which avoids the increased risks of morbidity and mortality associated with surgical intervention. 2 3

EID is the dissection in the intermuscular plane between the circular and longitudinal muscle layers of the muscularis propria. This technique allows for en-bloc resection of lesions with suspected DSI while preserving organ integrity and providing a comprehensive pathologic assessment of the retrieved specimen.

To explore the feasibility and outcomes of this endoscopic approach, we present a retrospective case series of EID in patients with suspected deep submucosal invasive T1 rectal cancers or severely scarred areas based on preinterventional assessment.

This case series aims to explore the potential role of EID in risk-adapted treatment strategies for early rectal cancer and to evaluate the feasibility of implementing this technique in a tertiary endoscopic center.

## Methods

Between April and October 2025, 16 consecutive EIDs were performed at two academic centers and retrospectively evaluated.

**Fig. 1:**
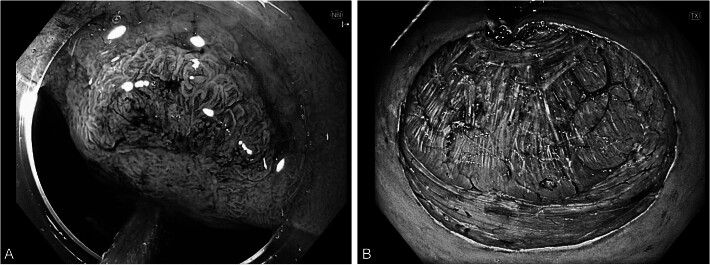
EID of a rectal lesion ( **A** ) Endoscopic view of the lesion prior to resection. ( **B** ) Postresection defect following EID with visible longitudinal muscle layer.

EID was performed for suspected deep submucosal invasive cancer (D-SMIC) based on advanced imaging (JNET classification, Paris classification), the presence of a nonlifting sign ( Fig. 1A ), or prior biopsy findings suggestive of DSI.

When D-SMIC was suspected, MRI was primarily performed in most cases for nodal staging and to exclude advanced local disease.

In cases of discrepancy between MRI findings and optical assessment regarding invasion depth, therapeutic decision-making was guided by endoscopic evaluation.

Patients were excluded from EID if baseline MRI showed tumor involvement at or above the sigmoid take-off or if there was evidence of malignant lymph nodes or adverse features such as extramural venous invasion or tumor deposits.

In two cases, a pre-interventional diagnosis of carcinoma was not assumed. Instead, a known neuroendocrine tumor and a vascular malformation were resected using EID.

EID was performed using a high-definition endoscope (CZ-1500) and the Olympus EVIS X1 System (Olympus Medical Systems, Tokyo, Japan). The lesion was marked with a safety margin of 5–10 mm in most cases, followed by submucosal injection of a lifting solution. After injection, a circular incision was performed, followed by preparation of the submucosal plane and subsequent careful dissection between the circular and longitudinal muscle layers ( Figs. 1B and Fig. 3 ). All lesions were resected en bloc. After resection, visible blood vessels were coagulated; the resection site was not closed in 10 of 16 cases. In six cases, the resection site was closed using the Olympus SutuArt hand-suturing system (Olympus, Tokyo, Japan).

**Fig. 2:**
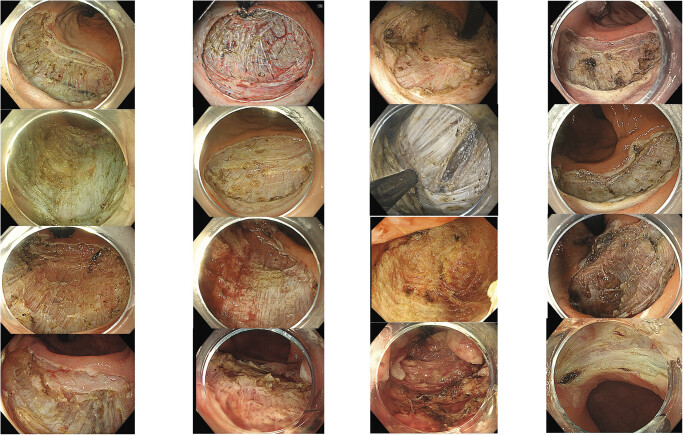
Postresection defects of all cases.

Fig. 2 shows a collage of all postresection defects following EID.

**Fig. 3:**
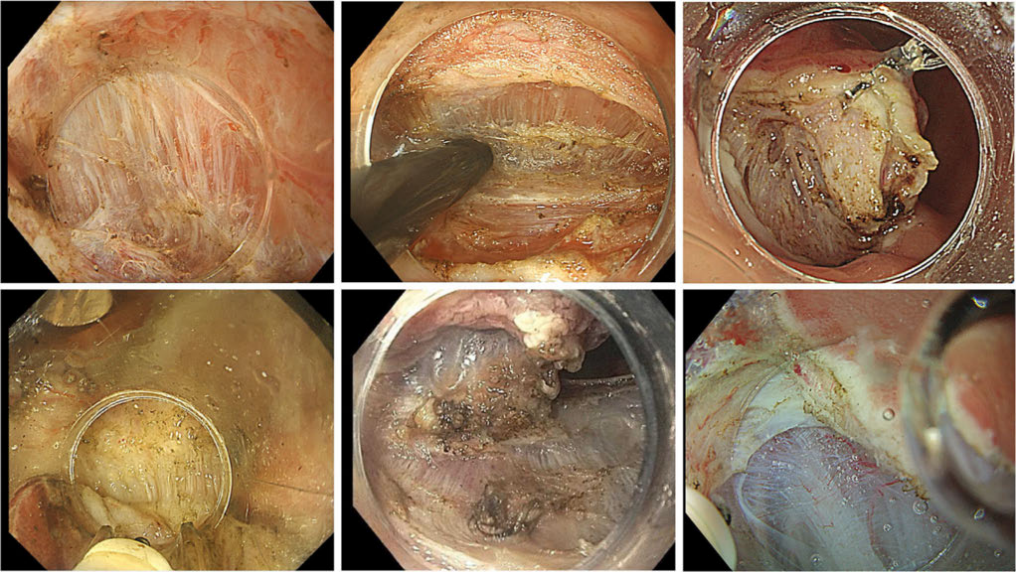
Intermuscular space during resection.

Serious adverse events (SAEs) were defined as clinically relevant, procedure-related events, including the need for endoscopic or surgical reintervention, blood transfusion, or hospital readmission due to endoscopy-related complications, as well as events resulting in sepsis, clinically relevant perforation, severe pain, or prolonged hospital or intensive care unit stay. In this retrospective analysis, SAE assessment was based on a systematic review of electronic medical records within 30 days after the procedure, including documentation of repeat interventions, hospital readmissions, clinical follow-up, analgesic requirements, and laboratory findings. Events managed endoscopically during the index procedure without subsequent clinical consequences were not classified as SAEs.

All EID procedures were performed by endoscopists with advanced expertise in endoscopic submucosal dissection (ESD). The operators considered the learning curve for EID to be manageable, with no substantial additional technical challenges compared with established ESD techniques. Delineation and dissection within the intermuscular plane were achievable in the majority of cases. Technical complexity was mainly encountered in previously treated lesions with marked fibrosis.

## Results

Sixteen patients underwent EID. Baseline patient characteristics are shown in Table 1 .

**Table 1:** Patient characteristics.

Characteristic	Total ( *n* = 16)
Age, years, mean	67
Sex	75% male/25% female
Estimated diameter of the lesion, median	26 mm (10–50 mm)
Lesion location, *n*	
Distal rectum (0–5 cm from anal verge)	9
Mid-rectum (6–10 cm from anal verge)	6
Proximal rectum (>10 cm from anal verge)	1
Anterior/posterior/between anterior and posterior/NA	2/4/2/8

Pre-interventional MRI was performed in eight cases. In the radiologic reports, no lesion was explicitly classified as T1; two cases were staged as T2, while the remaining reports did not provide a definitive distinction between T1 and T2 disease.

MRI findings, together with the corresponding histopathologic results and the pre-interventional endoscopic visual assessment, are summarized in Table 2 .

**Table 2:** Histopathologic and preinterventional staging.

Histopathologic staging	Pre-interventional MRI	JNET	Paris
pT1 (sm3), pNx, L0, V0, Pn0, G2, R1 (vertical margin), Bd 1	cT1/2, N0	2B/3	IIa
pT1 (sm3), pNx, L0, V0, Pn0, G2, R0, BD 1	cT2,N0,Mx	2B/3	IIa
Tubular adenoma, low grade	cT3, N0	2B	IIa
pT1 (sm2), Nx, L0, V0, Pn0, R0, G2, Bd1	cT1/2, N0	2B/3	0-Is-IIc
pT2, Nx, L0, V0, Pn0, G3, lokal R0, Bd1	cT2, N0	2B/3	IIa
rpTx, Nx, L0, V0, Pn1, G2, Bd 2, R1	No evidence of residual tumor, N0	2A/2B	IIa
pT1 (sm3), Nx, L0; V0, Pn0, G2, Bd1, R0	cT1/2, N0	2B	IIa
yPT1 (sm2), Nx, L0, V0, Pn0, G1, Bd1, R0	No evidence of residual tumor, N0	3	0-IIa+IIc

The average lesion size was 26 mm, with a range of 10–50 mm. The mean procedure duration was 139 minutes. All lesions were resected en bloc, with an overall R0 resection rate of 87.5% and 85.7% in lesions with D-SMIC ( Table 3 ).

**Table 3:** Case characteristics.

Patient	Lesion size*	Procedure time	JNET	Paris	Histopathologic staging
76y, m	20 mm	100 min	2B/3	IIa	pT1 (sm3), pNx, L0, V0, Pn0, G2, R0, Bd1
82y, f	10 mm	127 min	2B/3	IIa	pT1 (sm3), pNx, L0, V0, Pn0, G2, R1 (vertical margin), Bd 1
60y, f	50 mm	349 min	2B/3	IIa	pT1 (sm3), pNx, L0, V0, Pn0, G2, R0, Bd1
73y, m	30 mm	196 min	2B/3	0-Is-IIc	pT1 (sm2), pNx, L0, V0, Pn0, G2, R0, Bd1
61y, m	25 mm	135 min	2B/3	IIa	pT2, pNx, L0, V0, Pn0, G3, local R0, Bd1
70y, m	25 mm	95 min	2B	IIa	Adenoma with HGD
75y, f	30 mm	98 min	2B	IIa	Adenoma with LGD
75y, m	20 mm	82 min	-	-	NET, G1
53y, m	20 mm	89 min	2B	Is	Adenoma with HGD
71y, m	15 mm	185 min	2B	IIa	Adenoma with LGD (external pathology: high-grade dysplasia with transition to carcinoma in situ)
81y, m	30 mm	40 min	2B	0-IIa+IIc	pT1 (sm1), pNx, L0, V0, Pn0, G1, local R0, Bd1
81y, m	50 mm	148 min	2B	IIa	pT1 (sm3), pNx, L0, V0, Pn0, G2, local R0, Bd1
60y, m	25 mm	220 min	–	–	Vascular malformation
79y, m	20 mm	84 min	2A/2B	IIa	rpTx, pNx, L0, V0, Pn1, G2, Bd2, R1
49y, m	30 mm	218 min	2B	0-IIa+IIc	Adenoma with HGD
20y, m	20 mm	50 min	3	0-IIa+IIc	yPT1 (sm2), Nx, L0, V0, Pn0, G1, Bd1, R0

* Visual estimation in endoscopy.

Histopathologic examination confirmed low-grade adenocarcinomas with DSI, low TB, and no LVI in six cases (as exemplified in Fig. 5 ) and low-grade adenocarcinoma with superficial submucosal invasion, low TB and no LVI in one case. In one case, the rectal carcinoma had previously been treated with chemotherapy and radiotherapy. Histology revealed a low-grade T1 carcinoma with intermediate TB and perineural invasion. In this case, the resection was classified as R1.

In one case, histology confirmed a T2 carcinoma with PD (G3). Among the other seven cases, they showed tubular adenoma with HGD in three cases and LGD in another two cases. Furthermore, there was one neuroendocrine tumor and one vascular malformation removed with EID ( Table 3 ).

**Fig. 4:**
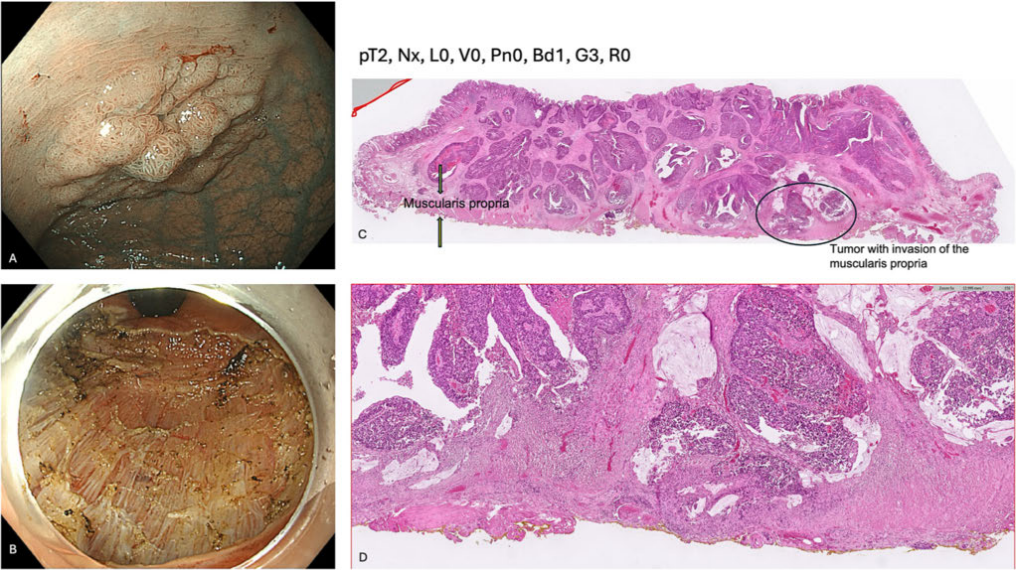
Histopathologic case demonstration. ( **A** ) Endoscopic view of the lesion prior to resection. ( **B** ) Postresection defect following EID. ( **C** ) Histologic specimen with the tumor indicated. ( **D** ) Magnified view of the tumor.

Histopathologic analysis demonstrated the presence of the circular muscle layer beneath the invasive front in all, but two specimens. In the R1 case, following partial conversion to endoscopic full-thickness resection, the invasive front extended beyond the circular muscle layer. In another case, histology revealed a T2 carcinoma with invasion into the muscularis propria; however, complete (R0) resection was achieved ( Fig. 4 ).

**Fig. 5:**
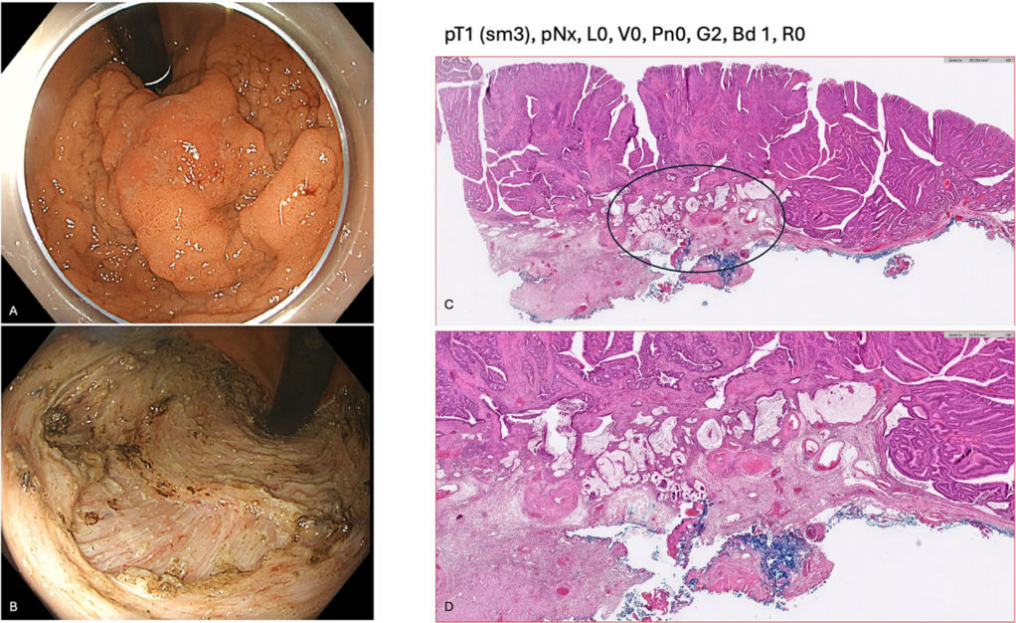
Histopathologic case demonstration. ( **A** ) Endoscopic view of the lesion prior to resection. ( **B** ) Postresection defect following EID. ( **C** ) Histologic specimen with the tumor indicated. ( **D** ) Magnified view of the tumor.

Two EID procedures were performed in individually managed, heavily pretreated patients following multidisciplinary tumor board decisions. In the first case, EID was undertaken after systemic therapy for hepatic metastatic and locally advanced rectal cancer as part of an individualized treatment strategy, with subsequent liver-directed therapy planned. The pre-interventional pelvic MRI showed no residual tumor or LNM. The procedure was technically feasible without major difficulty, and histology revealed an en bloc–resected deeply submucosal invasive carcinoma with negative margins (R0).

In the second pretreated patient, radiotherapy with curative intent was administered after an excellent response to initial systemic therapy for locally advanced rectal cancer.

**Fig. 6:**
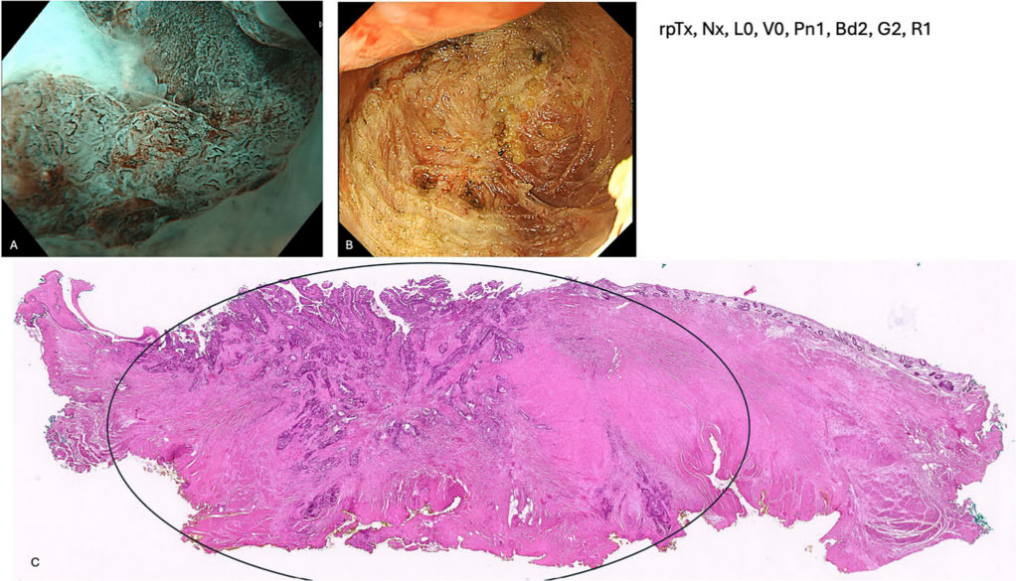
Histopathologic case demonstration. ( **A** ) Endoscopic view of the lesion prior to resection. ( **B** ) Postresection defect following EID. ( **C** ) Histologic specimen with the tumor indicated.

With no detectable tumor or lymph node involvement on pelvic MRI, EID was subsequently performed. The lesion extended to the anal canal and involved approximately one-quarter of the luminal circumference. Owing to pronounced posttreatment fibrosis and suspected deep tumor infiltration during the procedure, resection was performed partly as EID and partly as endoscopic full-thickness resection ( Fig. 6A,B ).

Histopathologic evaluation demonstrated tumor infiltration into the smooth muscle layer, with atypical glandular structures extending to the deep resection margin, resulting in an R1 resection status ( Fig. 6C ).

In both pretreated patients, pelvic MRI showed no detectable residual tumor; therefore, the indication for intervention was based solely on optical endoscopic assessment ( Table 2 ).

In one patient with confirmed D-SMIC, an R1 resection had to be presumed at the deep margin due to a specimen tear.

The specimen tear most likely occurred during retrieval of the EID specimen after complete resection. This patient subsequently underwent a surgical transanal full-thickness resection.

Histopathologic analysis of the surgical specimen revealed no residual tumor cells.

In one patient, histopathology revealed a T2 carcinoma with PD (G3). Following multidisciplinary tumor board recommendation, sphincter-preserving robotic intersphincteric rectal resection was performed; final histopathology demonstrated a post-EID ulcer without residual carcinoma or LNM.

During EID, dehiscence of the longitudinal muscle layer occurred in five cases. The maximum size of the transmural defect was approximately 10 mm. In four of these cases, the defect was small and closed using endoscopic clips, whereas the 10-mm defect was managed by complete closure of the resection site using the SutuArt system.

In cases with longitudinal muscle dehiscence, peri-interventional antibiotic therapy was administered and continued for an additional 2 days. Under this regimen, no delayed complications, including infection, pain, or perforation, were observed.

Peri-procedural bleeding was successfully controlled endoscopically using a Coagrasper (Olympus Medical Systems, Tokyo, Japan), and no patient required transfusion or re-intervention. No SAEs occurred in our cohort.

## Discussion

Although our case series is relatively small, the present findings are encouraging and provide further insight into the technical feasibility of EID in selected rectal lesions.

In this series, EID achieved technical success in all cases, with en bloc resection of all lesions.

In one patient, EID required partial conversion to endoscopic full-thickness resection owing to pronounced fibrosis after extensive prior therapy.

R0 resection rates in our series were encouraging. Across all cases, an R0 rate of 87.5% was achieved, and among D-SMIC lesions, the R0 rate was 85.7%. One case was classified as R1 because of specimen disruption, which precluded reliable margin assessment. However, no residual tumor was identified in the subsequent surgical specimen, suggesting that the lesion had most likely been completely resected endoscopically. Although this observation should be interpreted with caution, it indicates that the R0 resection rates for D-SMIC may have been underestimated in our cohort, rendering our findings broadly consistent with those reported in larger published series. 4

Beyond technical feasibility, the safety profile observed in our cohort is consistent with findings from previously reported EID series, which have described low rates of clinically relevant adverse events. 4 5 No postprocedural SAEs occurred, indicating a favorable risk profile in this selected population. Peri-interventional dehiscence of the longitudinal muscle layer was encountered in five cases; however, all defects were successfully managed endoscopically, either by clip closure or, in one larger defect, by endoscopic suturing. Notably, none of these events translated into clinically relevant delayed complications, underscoring the potential for effective endoscopic management even in technically demanding scenarios.

Current evidence supporting EID remains limited and is largely restricted to D-SMIC with no or one additional risk factor. 4 Available data suggest that careful patient selection is crucial, whereas more advanced tumors, particularly T3 lesions, require surgical management to maintain oncological safety. At the same time, overtreatment of superficial lesions without significant fibrosis should be avoided, as ESD remains an established and less invasive alternative.

Consequently, accurate pre-interventional diagnostics are essential for risk-adapted treatment decisions. Besides detailed optical assessment using endoscopic features such as Japan Narrow-band imaging (NBI) Expert Team (JNET), cross-sectional imaging is frequently incorporated to refine staging. However, MRI has recognized limitations in differentiating early T1 from more advanced T2 disease, which may lead to both over- and underestimation of tumor depth.

In our series, MRI-based staging showed both underestimation and overestimation when compared with final histopathology. In two pretreated patients, invasive disease was not detectable on pre-interventional imaging, whereas in other cases MRI overestimated tumor depth compared with final histopathology.

In routine clinical practice and across much of the existing literature, detailed differentiation between T1 and T2 stages on pre-interventional imaging is frequently not pursued or remains inconclusive. 6 In our cohort, three lesions ultimately classified as T1 on histopathology had not been clearly distinguished from T2 disease on pre-interventional MRI.

These findings illustrate the inherent limitations of radiologic staging in early rectal cancer and emphasize that MRI should be interpreted in conjunction with high-quality endoscopic assessment rather than as a stand-alone decision tool when selecting patients for EID.

Several limitations inherent to this study should be considered. The retrospective design, relatively small sample size, and heterogeneity of indications within the cohort limit the generalizability of our findings.

Patient selection relied heavily on pre-interventional assessment, which remains challenging, particularly with regard to differentiating T1 from T2 disease on MRI; as observed in our cohort, both underestimation and overestimation of tumor depth occurred, potentially introducing selection bias. Furthermore all procedures were performed by highly experienced endoscopists with extensive ESD expertise, which may limit the transferability of these results to less specialized settings. In addition, evidence regarding the role of EID after extensive pretreatment remains limited, and the small number of such cases in our series precludes firm conclusions.

## Conclusion

This retrospective small case series demonstrates that EID is a promising approach for the management of deeply submucosal invasive rectal carcinomas. In addition, the technique may be useful in the resection of adenoma recurrences or severely scarred areas.

The technique allowed for successful resection in all cases, with no significant complications.

Recognizing the potential of EID as an alternative to radical surgery, we are actively engaged in collecting and structuring additional data to strengthen the evidence base for its use. Ongoing analysis will aim to assess its outcomes in larger patient cohorts and refine patient selection criteria to maximize safety and effectiveness. As more data become available, EID could play a central role in an organ-preserving approach to T1 rectal cancer management, particularly for patients where pre-intervention assessment suggests a low risk of LNM.

This work underscores the importance of further research and collaboration to establish EID as a standard therapeutic option.
